# Factors That Affect Proliferation of *Salmonella* in Tomatoes Post-Harvest: The Roles of Seasonal Effects, Irrigation Regime, Crop and Pathogen Genotype

**DOI:** 10.1371/journal.pone.0080871

**Published:** 2013-12-04

**Authors:** Massimiliano Marvasi, George J. Hochmuth, Mihai C. Giurcanu, Andrée S. George, Jason T. Noel, Jerry Bartz, Max Teplitski

**Affiliations:** 1 Department of Soil and Water Science, University of Florida, Gainesville, Florida, United States of America; 2 Department of Statistics, University of Florida, Gainesville, Florida, United States of America; 3 Plant Pathology Department, University of Florida, Gainesville, Florida, United States of America; Cornell University, United States of America

## Abstract

**Main Objectives:**

Fresh fruits and vegetables become increasingly recognized as vehicles of human salmonellosis. Physiological, ecological, and environmental factors are all thought to contribute to the ability of *Salmonella* to colonize fruits and vegetables pre- and post-harvest. The goal of this study was to test how irrigation levels, fruit water congestion, crop and pathogen genotypes affect the ability of *Salmonella* to multiply in tomatoes post-harvest.

**Experimental Design:**

Fruits from three tomato varieties, grown over three production seasons in two Florida locations, were infected with seven strains of *Salmonella* and their ability to multiply post-harvest in field-grown tomatoes was tested. The field experiments were set up as a two-factor factorial split plot experiment, with the whole-plot treatments arranged in a randomized complete-block design. The irrigation treatment (at three levels) was the whole-plot factor, and the split-plot factor was tomato variety, with three levels. The significance of the main, two-way, and three-way interaction effects was tested using the (type III) F-tests for fixed effects. Mean separation for each significant fixed effect in the model was performed using Tukey’s multiple comparison testing procedure.

**Most Important Discoveries and Significance:**

The irrigation regime *per se* did not affect susceptibility of the crop to post-harvest proliferation of *Salmonella*. However, *Salmonella* grew significantly better in water-congested tissues of green tomatoes. Tomato maturity and genotype, *Salmonella* genotype, and inter-seasonal differences were the strongest factors affecting proliferation. Red ripe tomatoes were significantly and consistently more conducive to proliferation of *Salmonella.* Tomatoes harvested in the driest, sunniest season were the most conducive to post-harvest proliferation of the pathogen. Statistically significant interactions between production conditions affected post-harvest susceptibility of the crop to the pathogen. UV irradiation of tomatoes post-harvest promoted *Salmonella* growth.

## Introduction

Over the last decade, fruits and vegetables were among the foods most often linked to gastroenteritis outbreaks caused by enterovirulent strains of *E. coli* and non-typhoidal *Salmonella*, which resulted in thousands of hospitalizations and multi-million dollar losses to the horticultural food-crop industry [1]–[3]. Since 2006, at least sixteen salmonellosis outbreaks have been linked to tomatoes, cantaloupes, sprouts, cucumbers, mangoes, pine nuts, pistachios, peanut butter, papayas, and peppers in addition to mixed, frozen and processed foods containing plant products [4].

It is clear that *Salmonella* and other human pathogens can contaminate produce at any stage of the production cycle, farm to fork. The question of whether human enteric pathogens can utilize plants as alternate hosts [5], as well as its food safety implications, remain controversial. The rare, but consistent isolation of human pathogens from field-grown plants [6] and the observations that *Salmonella* and enterovirulent *E. coli* from plants can infect animals [7], [8] support the hypothesis that enterics can exploit plants as intermediate hosts. However, when avirulent *Salmonella* or *E. coli* surrogates were artificially introduced onto crops in large-scale field experiments, culturable cells of these pathogens declined [9]–[14], thus supporting the hypothesis that interactions between human pathogens and plants were transient or opportunistic. Nevertheless, in the field studies conducted in the Southeastern U.S.A., residual populations of human pathogens were capable of persisting for extended periods of time in the rhizosphere or within plant tissues [10], [11], [15]–[17].

Outbreaks of produce-associated gastrointestinal illness caused by human enteric pathogens have been sporadic, and their seemingly random nature argues for a “perfect storm” scenario [1]. Environmental conditions, multiple pre- and post-harvest production factors, genotype and physiological states of the crop and the pathogen, distribution routes, and exposure of the susceptible populations, etc. – may all converge to result in a major outbreak. Further complicating the “perfect storm” scenario is the fact that the transfer of the human pathogens onto plants and their proliferation within plants selects for microbial genotypes that are more adapted to the new environment [18]–[21]. To what extent each of these factors contributes to the “perfect storm” is not clear. A better understanding of the role of crop production practices that affect susceptibility of crops to human pathogens pre- and post-harvest could eventually result in a significant reduction of the number and/or severity of the produce-associated outbreaks.

Even though environmental persistence of *Salmonella* and enterovirulent *E. coli* in the field and under greenhouse conditions has been investigated [7], [10]–[12], [15]–[17], relatively little remains known about the impact of crop production practices on the susceptibility of plants to pathogens post-harvest. Recent evidence, however, points to the fact that farm management practices and environmental factors have profound effects on the persistence of enterics under field conditions and the susceptibility of crops to them [9], [22]. With this study we focused on the effects of irrigation practices on the susceptibility of tomato fruits to post-harvest proliferation of *Salmonella*.

The rationale for this study was based on models of plant disease, which suggest that varying the intensity of irrigation has significant effects on the susceptibility of crops to phytopathogens, as well as their persistence in the environment [23]. Even though *Salmonella* is not a plant pathogen, we hypothesized that similar mechanisms may underlie the interactions of this bacterium with plants. For the purpose of this paper, we define tomatoes that are more conducive to the proliferation of *Salmonella* as more “susceptible” to this pathogen. We further hypothesized that the impact of irrigation regimes on the susceptibility of vegetables to *Salmonella* post-harvest could be direct or indirect. For example, fruits harvested from plants exposed to excessive soil water near and during harvest period can become water congested or develop fruit surface cracking. Both water congestion and cracks are hypothesized to favor proliferation of *Salmonella* in tomatoes. Water stress is known to alter plant defenses [24], including those that have been shown to limit proliferation of human pathogens in plants [25], [26]. Furthermore, different levels of soil moisture can affect the composition of the rhizosphere microbiota, including those microbes with the biocontrol potential that induce systemic resistance. Over-irrigation can also promote the growth of phytophathogens, and this indirectly may favor proliferation of human pathogens [27]–[29]. However, before these mechanistic hypotheses are investigated in detail, it was important to first test whether there exists a relationship between the levels of irrigation and the susceptibility of crops to human pathogens.

## Materials and Methods

### Field Production Conditions

Seeds of tomatoes (cultivars Florida-47, Solar Fire, Bonny Best) were purchased from Siegers Seed Co. (Holland, MI) and Harris Co. (Rochester, NY). Transplants were raised in an environmental chamber on the University of Florida campus, and then planted in the field. Field experiments were conducted in three production seasons over two years in two geographic locations: Spring 2011 and Spring 2012 in Live Oak, FL (30°18′07.22′′; 82°53′58.865′′), and Fall 2012 in Citra, FL (29°24′37.84′′N; 82°10′12.14′′W). For a follow-up experiment, as indicated in the text, tomatoes were grown in Spring 2013 in Citra, FL.

Generally recommended practices for Florida tomato production were used for this research [30]. A cover crop (15 cm tall) of rye (*Secale cereale* L.) was rototilled in preparation for tomato production. At both sites, the soil tested high in phosphorus (P) and low in potassium (K) by the Mehlich-1 soil testing method [31], [32]. Pre-plant fertilizer (13N-2P-10K) was applied at 840 kg/ha to the bed area and rototilled into the soil prior to bedding and fumigating. The soil at each site was formed into raised beds with 1.5 m between the centers of adjacent beds and the soil was fumigated with a mixture of 50% methyl bromide: 50% chloropicrin to control soil-borne pests and weeds. Pre-emergence herbicides were applied carefully to the soil surface in the alleys between beds to control weeds. Drip irrigation tubing with emitters spaced 0.2 m apart applying 0.15 L/min/m^2^) was applied to the surface of the beds approximately 0.2 m to the side of the middle of the bed. Black polyethylene mulch was applied to the beds for the spring crops and silver-on-black for the fall. Three weeks after fumigation, tomato transplants were placed through holes in the mulch. Tomato plants were placed in single rows on the mulched bed with 0.4 m between plants in the row. The rows were spaced 0.3 m apart and the plants were spaced 0.3 m apart within rows. During the season, fungicides, bactericides, and insecticides were applied as recommended by field scouting and consistent with commercial tomato production practices.

A fertilizer injection system was set up to apply soluble fertilizer (N and K) in bi-weekly amounts to supplement the pre-plant fertilizer. 225 kg/ha of Nitrogen and 208 kg/ha of Potassium were applied per growing season. Irrigation was applied to maintain volumetric water content (measured by time domain reflectometry) at 10% [33]. Early in the season, one irrigation event of 30 minutes per day was satisfactory to maintain optimal soil moisture. Irrigation frequency was increased to two 30-minute runs per day as the crop developed and then finally to three 30-minute runs per day as the fruit matured. Two weeks prior to the onset of harvesting, the irrigation treatments were imposed. To achieve differences in the irrigation regimes, additional drip tubes were placed in the beds by threading them under the mulch with a string. One tube was used for the driest treatment, two tubes for the medium level, and three tubes for the wettest level. Three irrigation events of 30 minutes each were applied every day. The soil moisture targets for each treatment were 6, 10 and 12% volumetric water content. The yield was determined only for the tomatoes of commercially marketable sizes.

### Field Studies Ethics Statement

All experiments involving human pathogens were conducted following review and approval by the UF Office of Environmental Health and Safety. All field experiments were conducted on land owned by the University of Florida.

### Inoculations

Harvested tomatoes were brought into the lab and inoculated with *Salmonella* typically within 2–24 hours of the harvest. The inoculation procedure was chosen to mimic likely routes of post-harvest contamination (through shallow wounding and by depositing *Salmonella* on wounded surfaces) [34]. For the inocula, the type strain *S. enterica* sv Typhimurium ATCC14028 or *S.* Javiana ATCC BAA-1593, *S.* Montevideo LJH519, *S.* Newport C6.3, *S.* Braenderup 04E01347, 04E00783, 04E01556, which were linked to outbreaks of human salmonellosis were individually grown overnight at 37°C at 200 rpm in Luria-Bertani (LB) broth. They were then washed twice in phosphate-buffered saline (PBS), and the strains from the outbreaks were combined into a six-strain “cocktail”. The washed culture of *S.* Typhimurium 14028 and the outbreak cocktail were then further diluted in sterile water and 3 µl of the suspension (containing between 100 and 1,000 cells) were spotted into shallow (∼1 mm) wounds in the tomato epidermis. In addition to inoculating tomatoes with the cocktail of outbreak strains, in a subset of studies (as indicated in text), experiments were conducted with the outbreak strains individually.

For each inoculation, the dose was calculated based on the results of dilution plating. Infected tomatoes were incubated at 22°C for one week. Upon completion of the incubation, tomatoes were macerated in an equal volume of PBS using a stomacher (400 Circulator, Seward, Port St. Lucie, FL, USA) (200 rpm for 1 minute) and the suspension was plated onto a Xylose Lysine Deoxycholate (XLD) agar (Becton, Dickinson and Company, Franklin Lakes, NJ, USA) and incubated at 37°C overnight. Tomatoes (5–10 per sampling) harvested in the field, but not inoculated with *Salmonella* were similarly processed and tested on XLD to provide base-line assessment of the ability of the normal phytomicrobiota to grow on the selective medium. To account for the differences in tomato sizes and due to the fact that *Salmonella* does not uniformly colonize the interior tissues of the tomato fruit, an increase in proliferation was calculated by dividing the CFU recovered from fruits at final sampling by the CFU that were inoculated into each tomato. The ratios were further subjected to the log_10_ transformation. XLD plates on which there were no *Salmonella* colonies upon completion of the incubation were treated based on the rules of Most Probable Number (MPN) analysis [35], i.e. the most probable number, rather than a zero, was used for the calculations. This is a more conservative approach.

For the follow-up experiments in which the effects of surface UV disinfection were tested, tomatoes (cv. Amelia) were grown in Citra, FL in the Spring 2013 production season. Field grown tomatoes were washed in sterile tap water to collect rinsates for the re-inoculation experiments. UV disinfection was carried out for 10 min on the blossom end of the tomato, and 10 min on the stem-scar end in a Nuaire Class II Type A2 Biosafety hood under a Sylvania Germicidal Lamp (254 nm). After the treatment, tomatoes were quickly wiped with a paper towel wetted in 75% ethanol. Re-inoculation of the surface-disinfected tomatoes was conducted using collected rinsates. Rinsates were collected from 5 tomatoes harvested concurrently with those used in the experiments by rinsing them in sterile tap water and combining them. Tomatoes were submerged in the rinsates containing native epiphytic microbiota for 1 minute and air dried for ∼40 minutes in the biosafety hood prior to infections with *Salmonella* Typhimurium ATCC 14028. Control tomatoes were not re-inoculated following the surface disinfection.

### Water Congestion

Plugs (∼7 mm in diameter) were cut from tomato pericarps, and floated in sterile deionized water at room temperature. The increase in the mass of the pericarp fragments was recorded. An inoculum of *Salmonella* sv. Typhimurium 14028 was prepared as above, and infected into a shallow wound in the water-congested pericarp and incubated at 22°C for 24 or 48 hours. Upon completion of the incubation, samples were processed as above.

### Data Analysis

The experimental data were analyzed as a two-factor factorial split plot design, with the whole plot treatments arranged in a randomized complete block design. The whole plot factor was irrigation, with three levels, and the split plot factor was tomato cultivar, with three levels. Tomatoes of commercial size were harvested twice per season. Because the whole plot treatments were not randomized over the seasons, we used a split plot statistical design with repeated measures to analyze the data. Main effects, two-way, and three-way interaction effects were included in the model; higher order interactions were not considered in the model. The significance of the main effects, two-way and three-way interaction effects was tested using the (type III) F-tests for fixed effects. Mean separation for each significant fixed effect in the model was performed using Tukey’s multiple comparison testing procedure.

Data analysis was performed using SAS software. Specifically, we fitted the following linear mixed effects model for the split plot statistical design with repeated measures over seasons:
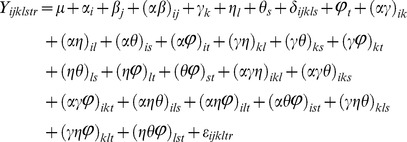
where µ is the overall mean, α_i_, γ_k_, η_l,_ θ_s_, and φ_t_ are the main effects of Irrigation, Cultivar, Strain, Maturity, and Harvest, β_j_, (αβ)_ij_, δ_ijkls_ are the random effects of Block, whole plot error, and split plot error, (αγ)_ik_, (αη)_il_, (αθ)_is_, (αφ)_it_, (γη)_kl_, (γθ)_ks_, (γφ)_kt_, (ηθ)_ls_, (ηφ)_lt_, and (θφ)_st_ are the two-way interaction effects, (αγη)_ikl_, (αγθ)_iks_, (αγφ)_ikt_, (αηθ)_ils_, (αηφ)_ilt_, (αθφ)_ist_, (γηθ)_kls_, (γηφ)_klt_, and (ηθφ)_lst_ are the three-way interaction effects, where β_j_ ∼ N(0,σ_β_^2^), (αβ)_ij_ ∼ N(0,σ_αβ_^2^), δ_ijkls_ ∼ N(0,σ_δ_^2^) are the independent random effects, and ε_ijklstr_ ∼ N(0,σ_ε_^2^) are the independent random errors.

The mixed effects linear model was fitted in SAS/GLIMMIX. We first identified the significant effects using the results of the F tests at a nominal P value of 0.05. Then, Tukey’s multiple comparison procedure (including the lines display) was carried out to separate the predicted means for the significant effects in the model. This post-hoc analysis enabled us not only to identify significant differences between various treatment means, but also to assess the treatments that were practically significant. Goodness-of-fit tests for the fitted model were conducted in SAS/UNIVARIATE by carrying out the Kolmogorov-Smirnov and Cramer-von Mises tests of normality of the studentized residuals. Since there was no strong evidence against the normality assumption of the studentized residuals, the statistical conclusions reported here are considered highly accurate and precise.

The analysis of the tomato yield data was performed using JMP software (SAS). Specifically, we fitted the mixed effects ANCOVA (Analysis of Covariance) model for the response variable Yield versus the categorical variables Irrigation, Cultivars, and Seasons. We included random effect of Blocks, Block*Irrigation and Block*Cultivar to comply with a split-plot with repeated measures experimental design. Only tomatoes of marketable size were included in this analysis.

## Results and Discussion

### The Effect of Irrigation Regimes on Tomato Yield

When designing this experiment, we aimed to test whether modifications in the irrigation regime, imposed within 2 weeks of harvest, will impact the susceptibility of tomatoes to post-harvest contamination with *Salmonella*. The goal of maintaining yields without strongly affecting them by the irrigation treatment was generally achieved (Table 1). Yield of tomatoes was strongly affected by the Cultivar and the Season. A three-way interaction Cultivar×Irrigation×Season was statistically significant (Table 1).

**Table 1:** Effects of independent variables (tomato cultivar, irrigation regime and season) on yield^1^.

Effects	F Ratio	Probability>F
Cultivar	26.2964	<.0001*
Irrigation	0.0734	0.9301
Season	76.5363	<.0001*
Cultivar×Irrigation	1.4191	0.2573
Cultivar×Season	43.5661	<.0001*
Irrigation×Season	1.4705	0.2086
Cultivar×Irrigation×Season	4.2922	<.0001*

1 Only marketable tomatoes were included in these analyses.

* Statistically significant interaction.

### Production Conditions and Susceptibility to Salmonella: Global Trends

**Figure 1:**
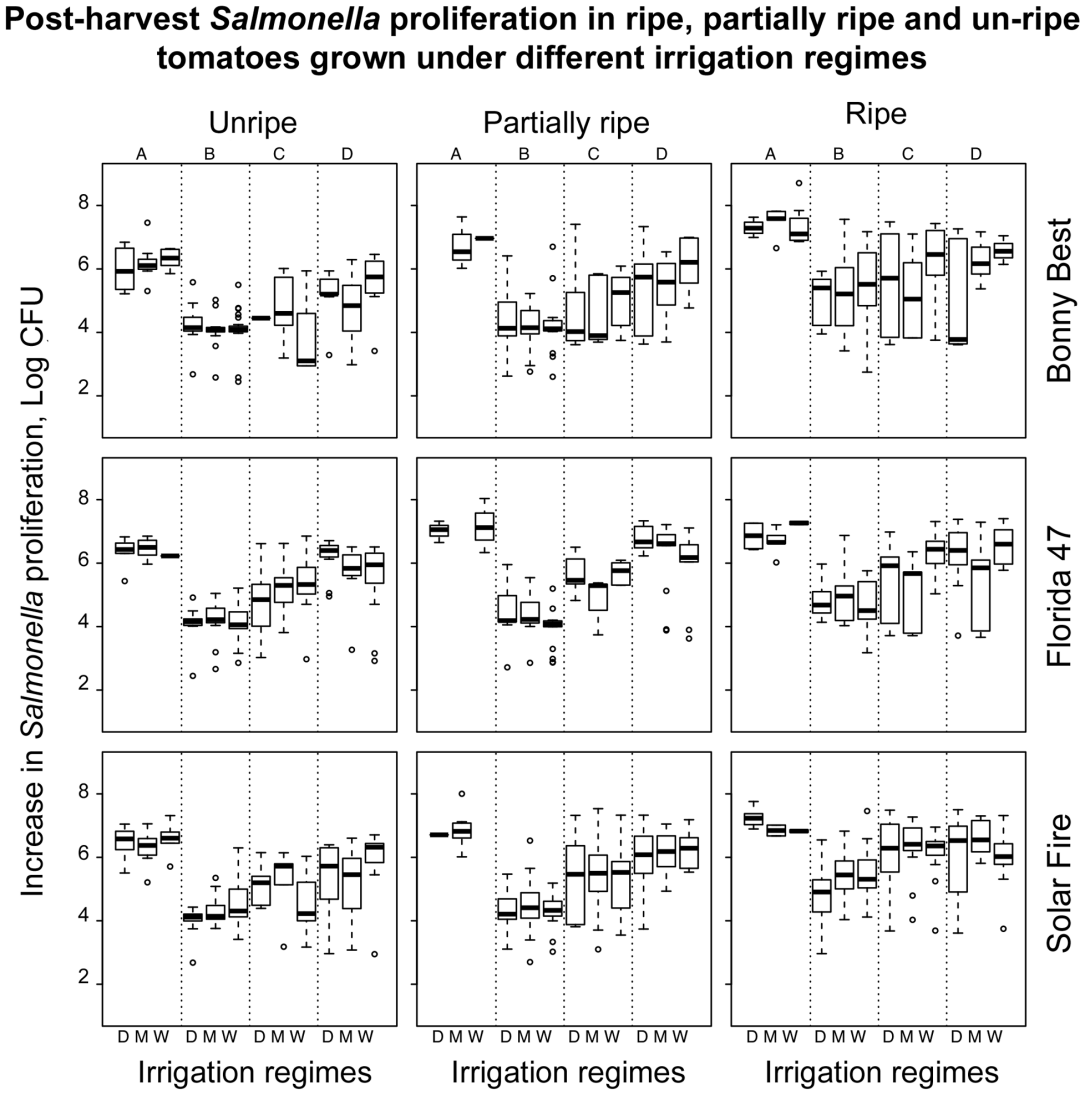
Post-harvest proliferation of *Salmonella* in ripe and un-ripe tomatoes grown under different irrigation regimes. Tomatoes (cultivars Bonny Best, Florida-47, and Solar Fire, indicated on the right y-axis) were grown under differential irrigation regimes: D (“dry”) = 6%, M (“medium”) = 10% (recommended for tomato production), W (“wet”) = 12% volumetric soil moisture contents were imposed within two weeks of the first harvest. Four independent samplings (A, B, C, D, top x-axis) were conducted: once in Spring 2011 and Spring 2012, and twice in Fall 2012. At each sampling, at least 55 tomatoes from each treatment were harvested and infected with ∼10^2^ CFU of *S.* Typhimurium 14028 or the cocktail of the six strains of *Salmonella* recovered from tomato-related human outbreaks. Upon completion of a 1-week incubation, *Salmonella* cells were recovered on Xylose Lysine Deoxycholate (XLD) agar and an increase in proliferation was calculated as Log (CFU/fruit_HARVEST_/CFU/fruit_INOCULUM_) and plotted on the y-axis for each tomato cultivar. Data for infections with both types of inocula are shown. “Unripe” tomatoes were mature green (stage 1 of the USDA Color Classification Requirements, http://ucanr.edu/repository/a/a=83755) at field harvest and *Salmonella* infection and were either breakers, turning, pink or light red (stages 2, 3, 4 or 5) upon completion of the 1 week-long incubation. “Partially ripe” refers for tomatoes that were mature green, breakers, turning or pink (stages 1, 2, 3 or 4) at field harvest and infection with *Salmonella*, but turned light red or red (stages 5 or 6) upon completion of the incubation. “Ripe” refers to tomatoes that were light red or red (stages 5 or 6) at field harvest and turned or stayed red during the incubation under the laboratory conditions. In box plots boxes include the lower and upper quartiles, thick lines within the box are the medians and whiskers indicate the degree of dispersion of the data. Outlier data are shown as dots.

As shown in Table 2 and Fig. 1, in neither of the growing seasons conducted at the two locations (North Florida and Central Florida), did differences in the irrigation regimes have any significant effect on the susceptibility of mature or immature tomatoes to post-harvest proliferation of *Salmonella*. Nevertheless, there were significant differences for the tomato genotype, strain of *Salmonella*, time of harvest, and maturity of the fruit at harvest. Two-way interactions between tomato genotype and irrigation regime, tomato genotype and time of harvest, *Salmonella* strain and time of harvest, tomato genotype and maturity were statistically significant (Table 2). Moreover, three-way interactions between tomato genotype, irrigation regime and time of harvest; tomato genotype, maturity and irrigation regime; tomato maturity, irrigation regime and time of harvest; and tomato maturity, *Salmonella* strain, and time of harvest were also statistically significant at 0.05 nominal level. The ANOVA (analysis of variance) table with the values of the F-tests and their corresponding p-values are in Table 2. These results are discussed below in detail.

**Table 2:** Type III F tests for the main, two-way and three-way interaction effects of irrigation levels, tomato cultivar, *Salmonella* strain, tomato maturity, and sampling on the susceptibility of the tomato to proliferation of *Salmonella*.

Effect	F value	Probability>F
Irrigation	0.7	0.5337
Tomato cultivar	4.02	0.0264*
Tomato cultivar×Irrigation regime	3.06	0.0393*
*Salmonella* strain	15.15	0.0001*
Irrigation regime×*Salmonella* strain	0.03	0.9663
Tomato cultivar×*Salmonella* strain	0.33	0.7199
Tomato cultivar×Irrigation regime×*Salmonella* strain	0.94	0.4401
Time of harvest^1^	289.04	<.0001*
Irrigation regime×Time of harvest	0.93	0.4736
Tomato cultivar×Time of harvest	4.9	<.0001*
Tomato cultivar×Irrigation regime×Time of harvest	2.84	0.0007*
*Salmonella* strain×Time of harvest	7.61	<.0001*
Irrigation regime×*Salmonella* strain×Time of harvest	0.28	0.9460
Tomato cultivar×*Salmonella* strain×Time of harvest	0.76	0.6049
Tomato maturity	71.31	<.0001*
Tomato maturity×Irrigation regime	0.29	0.8852
Tomato maturity×Tomato cultivar	4.13	0.0025*
Tomato maturity×Tomato cultivar×Irrigation regime	2.36	0.016*
Tomato maturity×*Salmonella* strain	0.34	0.7125
Tomato maturity×Irrigation regime×*Salmonella* strain	0.59	0.6690
Tomato maturity×Tomato cultivar×*Salmonella* strain	0.52	0.7183
Tomato maturity×Time of harvest	1.58	0.1506
Tomato maturity×Irrigation regime×Time of harvest	1.95	0.0256*
Tomato maturity×Tomato cultivar×Time of harvest	0.56	0.8751
Tomato maturity×*Salmonella* strain×Time of harvest	4.06	0.0005*

An asterisk (*) indicates statistically significant interactions between variables.

1 “Time of harvest” refers to the field sampling date (Spring 2011, Spring 2012 and twice in Fall 2012).

### Fruit Maturity Stage and Salmonella Proliferation

**Figure 2:**
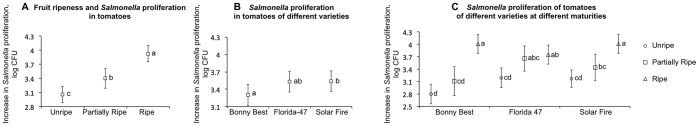
Effects of tomato genotype and fruit ripeness on proliferation of *Salmonella*. Tomatoes (cultivars Bonny Best, Solar Fire and Florida-47) were harvested in the field as for the commercial harvest, infected with 10^2^ CFU of *Salmonella* and incubated for a week. Maturity stages of the fruits at the time of infection with *Salmonella* were assessed using the USDA guide for tomato maturity. Tomato ripeness was assessed using USDA maturity chart. Tomatoes that were at stages 5 or 6 at field harvest were considered “ripe”, those that were harvested at stage 4 or below and then ripened during the experiment, were considered “partially ripe”, and those that were harvested at stage 3 or below and did not ripen beyond stage 5 during the experiment were considered “unripe”. An increase in *Salmonella* proliferation per fruit, relative to the initial inoculum is plotted. Medians are plotted, whiskers are standard errors. **A.** Effect of fruit ripeness as the main effect on *Salmonella* proliferation. **B.** Effect of the tomato genotype as the main effect on *Salmonella* proliferation. **C.** The effect of interactions between tomato genotype and maturity on *Salmonella* proliferation. Data from all samplings are included in each chart. Lower-case letters within the chart indicate groupings (p<0.05).

Under the field conditions, one of the strongest observed effects was the increased susceptibility of ripe fruit to post-harvest proliferation of *Salmonella* (Fig. 2). Strong statistically significant differences were observed in the proliferation of *Salmonella* in tomatoes harvested at different maturity stages. As shown in Fig. 2, final cells numbers of *Salmonella* were, on average 1 log higher in ripe tomatoes compared to the unripe tomatoes under the same conditions. In each season, there were samples in which *Salmonella* populations within red ripe tomatoes increased by at least 10^5^ from the initial dose of ∼10^2^ cells. Even though means of *Salmonella* proliferation in ripe and unripe tomatoes varied by no more than 2 logs across all seasons, the maximal proliferation of the pathogen in red tomatoes was almost always at least two logs higher than the highest cells numbers reached within unripe tomatoes. These observations are consistent with the reports that red tomatoes were significantly more conducive to proliferation of *Salmonella* than green tomatoes [36].

### The Role of Plant Genotype in Susceptibility of the Field Crop

Earlier studies documented differences in the ability of different crops and crop genotypes to support populations of enteric pathogens [9], [37], [38]. Furthermore, expression of specific *Salmonella* genes responded to the tomato genotype in general, and to the presence of certain tomato genes and metabolites in particular [39], [40]. Using Tukey-Kramer grouping of effects of cultivars in all seasons, fruits of tomato Bonny Best were found to be less conducive to *Salmonella* proliferation compared to the fruits of cv. Solar Fire (Fig. 2B). It is also important to note that when these differences were further dissected to establish two-way interactions (Cultivar×Sampling Time), the statistically significant differences between cultivars were observed within late and early Fall 2012 harvests (Table 3). Strong statistically significant differences in the proliferation of *Salmonella* were observed at different ripeness stages of each cultivar, and the magnitude of these differences was cultivar-dependent (Fig. 2C). The three-way interaction effects of Maturity×Cultivar×Irrigation were significant: the effects of the irrigation regime was significant for some of the Maturity×Cultivar groups; however, the Tukey mean separation for all LSMEANS did not identify Maturity×Cultivar groups for which the effects of Irrigation were significant (Table 4).

**Table 3:** Predicted means and Tukey mean separation (with letter groupings) for the two-way interaction effects of factors Cultivar and Sampling time with respect to susceptibility of tomatoes to proliferation of *Salmonella*.

Samplingtime	Cultivar	LSMEAN	Within-samplinggrouping(p<0.05)	Overallgrouping(p<0.005)
Spring 2011	Florida47	4.6397	A	A
Spring 2011	Bonny Best	4.5960	A	A
Spring 2011	Solar Fire	4.4769	A	AB
Spring 2012	Florida47	2.4019	A	AB
Spring 2012	Bonny Best	2.3736	A	AB
Spring 2012	Solar Fire	2.2046	A	B
Early Fall 2012	Florida47	3.1227	A	C
Early Fall 2012	Bonny Best	3.0064	A	CD
Early Fall 2012	Solar Fire	2.5385	B	DE
Late Fall 2012	Florida47	4.2849	A	E
Late Fall 2012	Bonny Best	4.1647	A	E
Late Fall 2012	Solar Fire	3.7220	B	E

**Table 4:** The effect of independent variables (tomato cultivar, ripening stage, irrigation regime) on the susceptibility of the crop to proliferation of *Salmonella*.

Maturity×Cultivar×Irrigation Regime	EstimateLS-Means	ANOVA
Ripe×Bonny Best×Over-irrigated	4.3889	A
Ripe×Solar Fire×Optimal	4.2016	A
Ripe×Bonny Best×Optimal	4.0372	BAC
Ripe×Solar Fire×Under-irrigated	4.0031	BAC
Ripe×Florida-47×Over-irrigated	3.9709	BAC
Ripe×Florida-47×Under-irrigated	3.9525	BAC
Ripe×Solar Fire×Over-irrigated	3.8344	BAC
Partially ripe×Florida-47×Under-irrigated	3.7491	BDAC
Partially ripe×Solar Fire×Optimal	3.6462	BDAC
Partially ripe×Florida-47×Optimal	3.6121	BDAC
Partially ripe×Florida-47×Over-irrigated	3.6107	BDAC
Ripe×Bonny Best×Under-irrigated	3.6093	BDAC
Partially ripe×Solar Fire×Over-irrigated	3.4177	BDAC
Partially ripe×Bonny Best×Over-irrigated	3.4072	BDAC
Ripe×Florida-47×Optimal	3.3303	BDAC
Un-ripe×Solar Fire×Over-irrigated	3.2666	BDAC
Partially ripe×Solar Fire×Under-irrigated	3.2552	BDAC
Un-ripe×Florida-47×Over-irrigated	3.2454	BDC
Un-ripe×Florida-47×Optimal	3.1809	BDC
Un-ripe×Solar Fire×Optimal	3.1726	DC
Un-ripe×Florida-47×Under-irrigated	3.1535	DC
Broker×Bonny Best×Optimal	3.0800	DC
Un-ripe×Solar Fire×Under-irrigated	3.0765	DC
Broker×Bonny Best×Under-irrigated	2.8482	DC
Un-ripe×Bonny Best×Optimal	2.8184	D
Un-ripe×Bonny Best×Over-irrigated	2.7972	D
Un-ripe×Bonny Best×Under-irrigated	2.7811	D

### The Role of the Salmonella Genotype in Proliferation within Tomatoes

**Figure 3:**
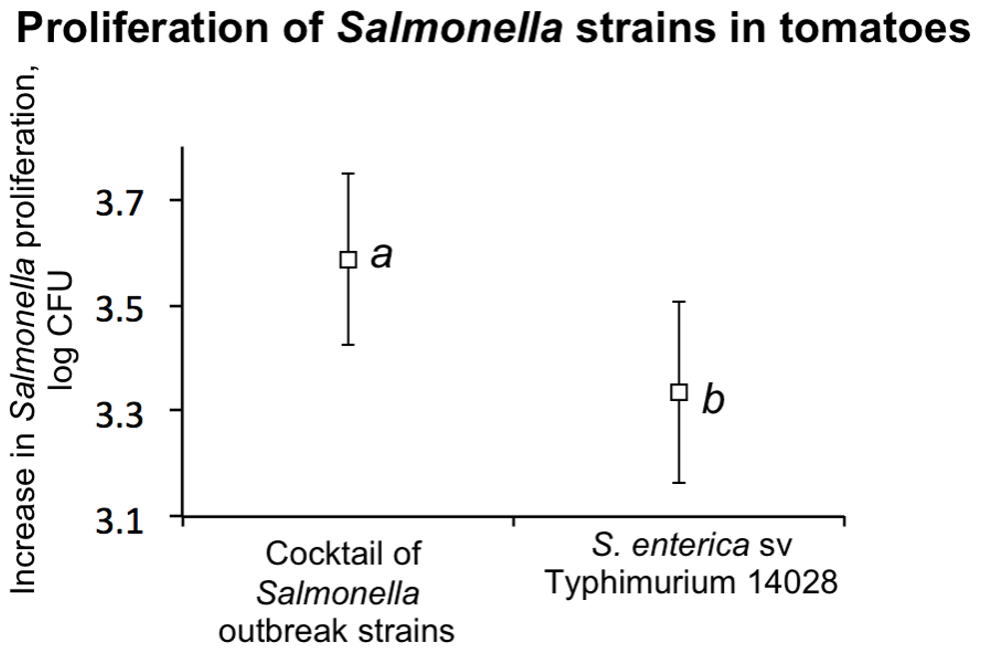
Serotype-level differences in *Salmonella* proliferation in tomatoes. Post-harvest proliferation of the type strain of *S.* Typhimurium ATCC 14028 and a cocktail of six strains of *Salmonella* (*S.* Javiana ATCC BAA-1593, *S.* Montevideo LJH519, *S.* Newport C6.3, *S.* Braenderup 04E01347, 04E00783, 04E01556) recovered from tomato-linked outbreaks of salmonellosis was tested in mature and immature tomatoes of three varieties and in peppers. Pairwise comparisons were conducted using the Student’s *t* test. Lower case letters indicate groupings (p<0.05).

The infections were conducted using either a monoculture of the type strain of *S. enterica* sv. Typhimurium ATCC14028 (originally isolated from a diseased animal) or using a cocktail of the *Salmonella* strains (Newport, Javiana, Braenderup, and Montevideo) linked to human salmonellosis outbreaks resulting from the consumption of tomatoes. The rationale for this experimental set-up is provided in the Framework for Developing Research Protocols for Evaluation of Microbial Hazards and Controls During Production [41]. Briefly, it is hypothesized that when testing effects of each individual strain represents a logistical burden, a cocktail of the strains recovered from outbreaks associated with a particular commodity or an environmental source would represent a suitable approximation of the behavior of a microorganism that is most fit under the conditions of interest [41]. Consistent with this postulate, a cocktail of the outbreak strains was capable of growing to the higher final populations numbers within field grown tomatoes (Fig. 3).

When infections were carried out with individual strains, within green or pink tomatoes, *S.* Newport reached the highest final cell count (log 10.20±0.08 and 9.94±0.02, respectively), with *S.* Montevideo and Javiana reaching significantly (p<0.05) lower final cell numbers of (log 9.66±0.16 and 9.73±0.02 for *S.* Montevideo green and pink, and log 8.59±0.06 and 9.71±0.02 for *S.* Javiana). In green and pink tomatoes, *S.* Typhimurium ATCC14028 reached numbers (log 9.98±0.08 and log 9.93±0.02) that were significantly (p<0.05) higher than those reached by *S.* Montevideo and *S.* Javiana, and generally lower than the final cell counts reached by *S.* Newport. This observation is similar to reports of differences in the proliferation of the *Salmonella* strains in plant tissues, including tomato fruits [36], [42]. At least in part, this observation could be due to the non-*rdar* phenotype of *S.* Newport and the rapid evolution of non-*rdar* mutants of *S.* Typhimurium ATCC14028. The non-*rdar* phenotypes have been shown to increase fitness of *Salmonella* strains in tomatoes and were associated with the strains recovered from produce [20].

### Fruit Water Congestion and Salmonella Proliferation

It was hypothesized that over-irrigation may result in fruit water congestion. Therefore, under laboratory conditions, we tested whether congesting tomato pericarp sections artificially affects proliferation of the pathogen. Water congestion of pericarp sections excised from green tomatoes resulted in a 5-fold increase of *Salmonella* proliferation after 24 hours, and a 10-fold increase after incubation for 48 hours (*p*<0.0001). On average, *Salmonella* sv. Typhimurium 14028 reached log_10_ = 8.33/wound in water congested pericarp sections of green tomatoes. Under similar conditions, water congestion of pericarp fragments from red tomatoes did not increase the ability of *Salmonella* to multiply within them. *Salmonella* reached, on average, log_10_ = 6.75 cfu/wound in control pericarp sections excised from red tomatoes, and log_10_ = 6.82 cfu/wound in water congested sections after a 24-hour incubation. Therefore, even though none of the field-tested irrigation regimes resulted in water congested fruit, production conditions or post-harvest treatments that cause water congestion could increase the proliferation of the pathogen within tomatoes. The mechanism by which water congestion favored proliferation of *Salmonella* within tomato pericarps is not yet clear: it could be due to a number of physical and chemical changes experiences by the water congested fruit.

### Plant Disease Pressure and Proliferation of Salmonella

Previous studies have clearly demonstrated that *Salmonella* proliferates to significantly higher numbers in the presence of plant pathogens or plant lesions [27], [28], [43]–[47]. However, tomatoes with obvious signs of spoilage are likely to be discarded prior to reaching the consumers.

**Figure 4:**
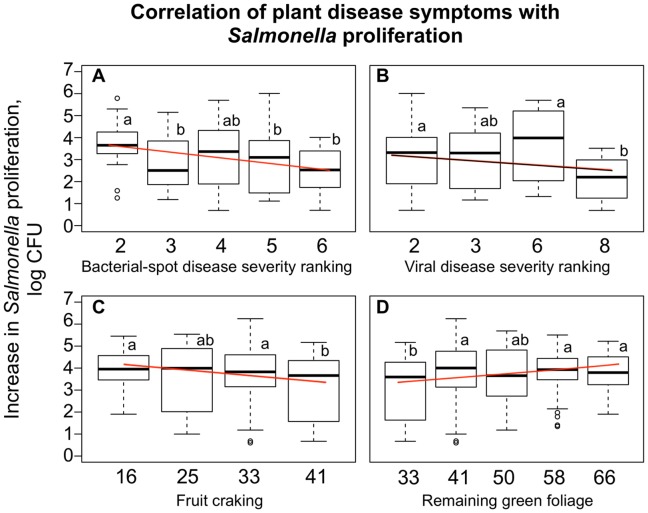
Correlation of plant disease symptoms with susceptibility to *Salmonella*. Bacterial spot disease severity and viral symptoms in tomato cv. Bonny Best were ranked based on a 1 10 scale, where 1 is a blemish-free fruit, and 10 is severely damaged fruit (Panels A, B). *Xanthomonas, Pseudomonas* and *Sphingomonas* were recovered from the bacterial spot lesions, viral symptoms were consistent with infections by Tomato Yellow Leaf Curl group virus. Fruit cracking and defoliation (as percentage of remaining green foliage) of the tomato cv. Solar Fire was assessed as an indirect measure of plant health (Panels C, D). The survey was conducted in the third production season in Fall 2012, Citra, FL. Double-blind disease severity rankings were conducted by two scientists independently on different days during the growing season; *Salmonella* proliferation and correlation analyses were conducted by a third scientist. Proliferation of *Salmonella* in blemish-free tomatoes harvested from these plants was tested. Box plots indicate relative proliferation of the type strain and the outbreak strains in blemish-free tomatoes from the corresponding treatments. The fitted regression lines are displayed in red and the P values (α = 0.05) of the F tests for the slope parameters are: Panel A = 0.0024, Panel B = 0.2508, Panel C = 0.0078 (not significant), and Panel D = 0.0462.

With this study we tested whether there is a correlation between disease pressure and susceptibility to *Salmonella* proliferation of blemish-free tomatoes harvested from plants otherwise showing symptoms of bacterial spot, viral infection, fruit cracking and the remaining green foliage. These surveys were conducted on the crops grown in Fall 2012. Tomato plants with the symptoms of the bacterial spot were submitted to the University of Florida Plant Pathology clinic, and *Xanthomonas spp, Pseudomonas spp,* and *Sphingomonas spp* were recovered from the lesions. Plant disease severity rankings were double-blind and were conducted by two scientists independently, on different dates. The remaining green foliage was used as an indirect measure of plant health and it negatively correlated with the disease severity (Panel C, Fig. 4), regardless of the cause.

Curiously, there was a statistically significant trend suggesting that blemish-free tomatoes harvested from plants with the most severe disease symptoms were less conducive to the proliferation of *Salmonella* (Fig. 4A, C). The severity of viral symptoms did not correlate strongly with the increased susceptibility of the fruit to *Salmonella* (Fig. 4B).

The mechanism responsible for this observed effect is not yet clear. There are at least two possibilities: (1) blemish-free fruits from otherwise diseased plants may contain elevated levels of plant defense compounds, which may reduce proliferation of *Salmonella*; or (2) asymptomatic fruits may contain microbiota that is less conducive to the proliferation of this organism. The synergistic and antagonistic effects of phytomicrobiota on proliferation of human pathogens are well documented [29], [48].

### Seasonal Effects

**Figure 5:**
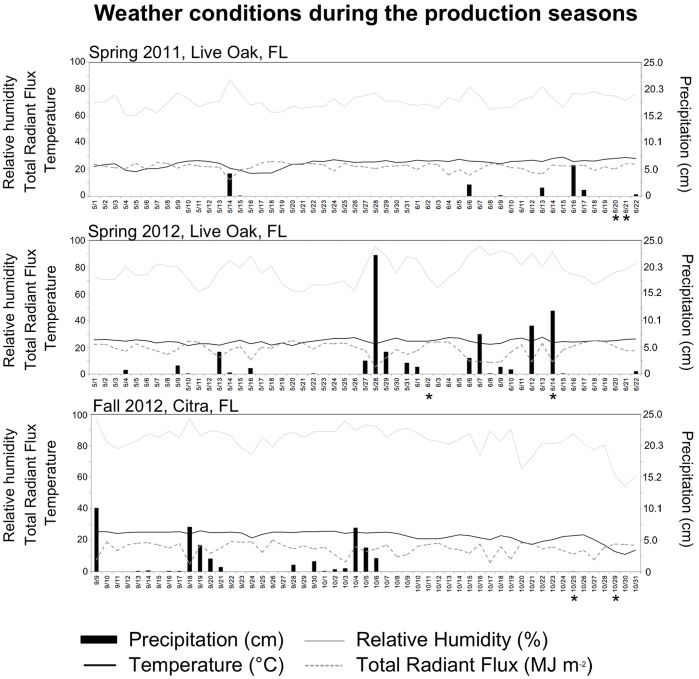
Weather conditions during the production seasons. Relevant weather data (as suggested by [41] was obtained from Florida Automated Weather Network (fawn.ifas.ufl.edu). Precipitation (cm) is shown as grey bars (right y-axis), relative humidity (dotted line), total radiant flux (dashed line), temperature (solid black line) are shown on the left y-axis. Days are plotted on the x-axis. Dates of harvest are shown with an asterisk.

Aside from maturity, seasonal effects were most obvious (Table 2, Fig. 1). Strong seasonal variability was also noted in the field studies with a strain of enterohemorrhagic *E. coli* and spinach [9]. *Salmonella* proliferation was the highest in fruits harvested in Spring 2011 (Live Oak, FL), the lowest during sampling in Spring 2012 (Live Oak, FL) and intermediate during the early and late Fall 2012 samplings (Citra, FL) (Fig. 1). Because tomatoes that were the most and the least conducive to *Salmonella* proliferation were harvested in the same geographic location, the field site was not solely responsible for these differences. Weather conditions within a month prior to harvests were different in each of the experimental seasons (Fig. 5) and weather parameters suggested as consequential to the proliferation of human pathogens in the field [41] are discussed below. Average daily temperatures in Spring 2011, Spring 2012 and Fall 2012 were 26.8°C, 24.8°C, 21.2 and 20.3°C, respectively. It is of note that the last harvest in Fall 2012 was immediately preceded by a decrease in temperature to 1.6°C. Even though the signs of chilling injury were not observed on tomatoes, exposure to low temperatures may be at least in part responsible for the differences in the susceptibility of tomatoes harvested in October 2012 within a week of each other. Average relative humidity during these production seasons was 69.5, 74.04, 86.9 and 85.7%. Total precipitation was 9, 33.1, 0.06 and 0.06 (cm m^−2^). Average total radiant flux was 21.55, 17.6, 14.28 and 13.6 (MJ m^−2^) in these production seasons. Therefore, the season in which the tomato crops were the most conducive to proliferation of *Salmonella* was with the low cloud cover and relative humidity, and few precipitation events. Of note, an inverse correlation between the rainfall and the prevalence of *Salmonella* in the samples collected in a major vegetable-growing region of California has been reported [49]. In our study, the amount of precipitation per se was probably inconsequential considering the fact that in none of the three seasons water supplied by drip irrigation had a significant effect on the susceptibility of the harvested fruit to proliferation of *Salmonella*. Furthermore, in interpreting these results, it is important to consider that *Salmonella* did not experience any of these conditions in the field, and the seasonal effects on the post-harvest proliferation of *Salmonella* within tomatoes are indirect.

Several hypotheses can be formulated to explain these results. For example, acidity and sugar composition may be responsible for the observed differences. However, total acid composition was shown to not vary seasonally in tomato fruits, while seasonal effects on the tomato fruit sugar content (including sucrose, which is not typically metabolizable by *Salmonella*, fructose and glucose) have been reported [50]. Even if concentrations of sugars vary in plant tissues, it is not certain that this will affect growth of *Salmonella*: studies of its metabolism in animal tissues revealed that *Salmonella* is an efficient scavenger, which concurrently utilizes multiple nutrients and most metabolic mutants were not defective in host colonization [51], [52]. Therefore, even though levels of sugars (and, potentially, other carbon and nitrogen sources metabolizable by *Salmonella*) may vary depending on the environmental conditions associated with different seasons, they are not likely to explain the observed seasonal differences. Therefore, we pursued an alternative hypothesis that attempted to link environmental conditions (high solar irradiation, low humidity), observed in the season when tomatoes were most conducive to *Salmonella* proliferation and a potential involvement of the plant microbiota in this interaction.

### Effect of UV Irradiation and Normal Plant Epimicrobiota on Salmonella Proliferation in Tomatoes

**Figure 6:**
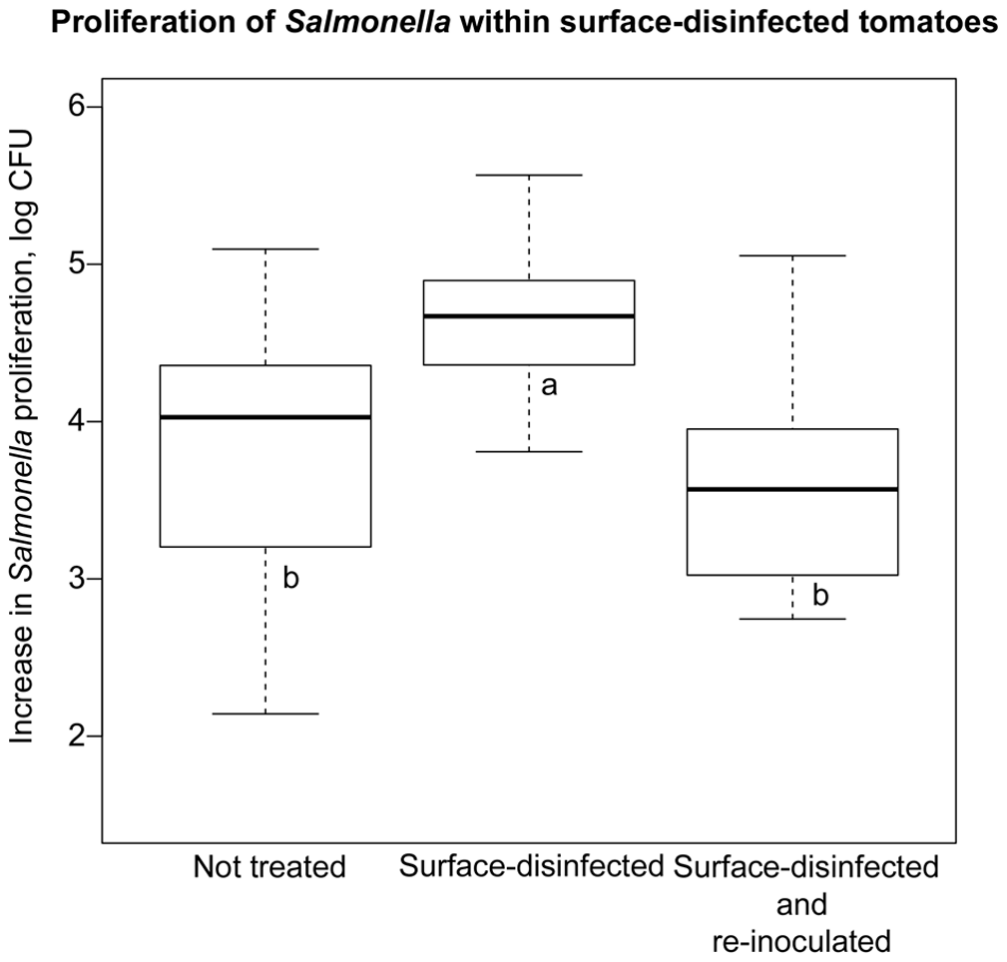
Proliferation of *Salmonella* in surface disinfected tomatoes. Tomatoes (cv. Amelia) were grown in Citra, FL in the Spring 2013 production season. Rinsated were collected from field-growth tomatoes in sterile tap water, and these were used to re-inoculate some of the tomatoes. UV disinfection was carried out for 10 min on the blossom end of the tomato, and 10 min on the stem-scar end in a Nuaire Class II Type A2 Biosafety hood under a Sylvania Germicidal Lamp (254 nm). Control tomatoes were not re-inoculated following the surface disinfection. All tomatoes were then infected with *Salmonella* Typhimurium ATCC 14028.

The rationale for the hypothesis that the seasonal variation in susceptibility of tomatoes to *Salmonella* proliferation could be due to the epiphytic microbiota is based on field studies, which demonstrated that the composition of the plant epimicrobiota was subject to strong seasonal effects [13], [53], and that phytomicrobiota can exert both agonistic and antagonistic effect on human pathogens *in planta* [48], [54], rev. [29]. Furthermore, the complexity of the phytomicrobiota correlated with the ability of the pathogenic *E. coli* to persist on plant surfaces [13]. Therefore, in laboratory experiments, we exposed field-grown tomatoes to UV irradiation and then either re-inoculated them (or not) with the rinse containing the original microbiota prior to the infections with *Salmonella*. As shown in Fig. 6, UV irradiation of fruit surfaces led to an increased proliferation of *Salmonella* within fruits. Re-inoculation of the irradiated surfaces with the water rinse containing native microbiota led to a reduction in the ability of this pathogen to multiply within tomato fruits. Therefore, it is possible that at least in part, the correlation between the driest, sunniest production seasons and the increased proliferation of *Salmonella* within tomatoes could be due to changes in the epiphytic microbiota. The mechanisms by which plant epiphytes inhibit proliferation of *Salmonella* within tomatoes are not immediately clear. However, because *Salmonella* was inoculated onto shallow wounds in tomato fruit epidermis, it was possible that the inoculated pathogen was in direct contact with the native microbes within wounds.

## Conclusions

Artificially congesting green tomato fruits with water led to ∼1 log increase in *Salmonella* proliferation. However, changes in the irrigation regime imposed within 2–4 weeks of harvest had no observable effect on the susceptibility of tomato fruits to post-harvest proliferation of *Salmonella* in the Spring production seasons of 2011 and 2012, and in early and late Fall 2012 in two geographic locations using three cultivars of tomato. Maturity of the fruit and seasonal variability were the strongest factors that correlated with the susceptibility of the crops to *Salmonella*. In some seasons, tomatoes of the cultivar Bonny Best were less conducive to *Salmonella* proliferation compared to cultivars Solar Fire or Florida-47. The effects of Maturity×Cultivar×Irrigation were significant and the effects of the irrigation regime were significant for some of the Maturity×Cultivar groups; however, the Tukey mean separation for all LSMEANS did not identify Maturity×Cultivar groups for which the effects of Irrigation is significant. Curiously, tomatoes harvested in the driest, sunniest of the seasons were the most conducive to *Salmonella* proliferation. Blemish-free tomato fruits harvested from tomatoes displaying symptoms of bacterial spot were less conducive to *Salmonella* proliferation.

## Acknowledgments

We thank C. Chen, M. Carrazana, E. Gause, M. Farias, A. Aruca, M.H. Moraes, K. Jenkins, and D. Lucas for their help with tomato processing during harvest.
